# A randomized controlled trial into the effectiveness of a mobile health application (SAM) to reduce stress and improve well-being in autistic adults

**DOI:** 10.1177/13623613251346885

**Published:** 2025-06-26

**Authors:** Kirsten L Spaargaren, Yvette Roke, Sander M Begeer, Annemieke van Straten, Heleen Riper, Kirstin Greaves-Lord, Anke M Scheeren

**Affiliations:** 1 Vrije Universiteit Amsterdam, The Netherlands; 2 Amsterdam Public Health Research Institute, Mental Health, The Netherlands; 3 GGz Centraal, The Netherlands; 4 Amsterdam University Medical Center, The Netherlands; 5 Lentis Psychiatric Institute, The Netherlands; 6 University of Groningen, The Netherlands

**Keywords:** adults, application, autism, mental well-being, mHealth, perceived stress

## Abstract

Autistic adults experience more daily stress compared to non-autistic adults. However, stress recognition is often delayed, potentially leading to chronic stress and significant (mental) health risks. We evaluated the effectiveness of Stress Autism Mate (SAM), a stress-monitoring mobile health application co-created with autistic individuals. We ran a two-arm, parallel, superiority randomized controlled trial. Autistic adults (*n* = 214) (mean age: 50.5 years; 66.4% female) were randomized to 1-month SAM usage or waitlist control. Participants self-reported on primary (Perceived Stress Scale; Warwick-Edinburgh Mental Wellbeing Scale), and secondary outcomes (Coping Self-Efficacy Scale) at baseline and 4 weeks later, after which the control group could start using the app. Hierarchical multiple regression analyses revealed that the intervention group had significantly lower perceived stress (*d* = 0.27), greater mental well-being (*d* = 0.35), and improved coping self-efficacy (*d* = 0.16) compared to the control group after 1 month. No intervention effect moderators could be identified. More frequent app usage was significantly associated with a stronger decrease in perceived stress (*r* = −0.293). However, 42.9% of app users indicated increased stress from app use, warranting further investigation. Our results suggest that SAM may be effective in reducing perceived stress and improving mental well-being for some autistic individuals. Further refinement and investigation are recommended.

**Lay abstract:**

Autistic adults often experience more daily stress than non-autistic individuals, but they may not always recognize this stress timely, which can lead to long-term health problems. To help address this, we tested an app called Stress Autism Mate (SAM), which was designed together with autistic individuals to help autistic adults monitor and manage their stress. In our study, 214 autistic adults (average age: 50.5 years; 66.4% female) were randomly assigned to either use the app for 1 month or wait before using it. We asked participants to complete surveys before and after the 1-month period to measure changes in their stress levels, mental well-being, and confidence in coping. Results showed that participants who used the app reported lower stress, improved mental well-being, and better coping skills compared to those who did not use the app. We also found that the more often someone used the app, the more their stress levels decreased. However, 42.9% of users felt more stressed using the app, which needs further study. Overall, our findings suggest that SAM can help reduce stress and improve well-being for some autistic adults, but more work is needed to improve the app and better understand its effects.

*Trial registry* ISRCTN Registry: ISRCTN17394910.

Adults on the autism spectrum consistently report enhanced perceived stress compared to non-autistic adults (Hirvikoski & Blomqvist, 2015; McQuaid et al., 2022; van der Linden et al., 2022). The level of “perceived stress,” here defined as the tendency or degree to which one appraises life events as stressful (Cohen et al., 1983), is determined by the balance between perceived demands and personal resources. Characteristics of autism, including differences in sensory processing, social interaction and communication, and rigidness, are thought to predispose autistic individuals to experience more stress (Gillott & Standen, 2007; Groden et al., 2006; Hirvikoski & Blomqvist, 2015).^1^ Moreover, autistic individuals are found to encounter more frequent daily stressors and more stressful life events than non-autistic adults (Bishop-Fitzpatrick et al., 2017; Moseley et al., 2021). In addition to these stressors, autistic individuals may also experience minority stress, resulting from their marginalized social status, discrimination, and internalized stigma (Botha & Frost, 2020). Apart from its detrimental effects on mental and physical health, chronic stress has been linked to an increase in autistic traits and co-occurring symptoms such as anxiety or depression (Fuld, 2018; Rosen et al., 2018), reduced independence, and poorer subjective quality of life of individuals with autism (McQuaid et al., 2022). This study examined the effectiveness of a stress-monitoring app in reducing perceived stress and improving mental well-being and coping self-efficacy in autistic adults.

When the demands placed on an individual are evaluated as overwhelming, that is, exceeding an individual’s resources, coping mechanisms are required to manage (Lazarus & Folkman, 1984). Autistic individuals on average have poorer stress regulating abilities than non-autistic individuals (Evers et al., 2023; Hirvikoski & Blomqvist, 2015; Muniandy et al., 2022). Importantly, to prevent stress from becoming chronic by employing effective coping, stress needs to be recognized timely. Yet, this timely recognition may be hampered in autistic individuals, as they are faced with interoceptive difficulties, that is, difficulties perceiving one’s own bodily states, including stress (Shah et al., 2016; Williams et al., 2023). Although the number of individuals with autism struggling with interoception specifically is unclear, sensory processing differences overall are one of the most prevalent symptoms of autism, reported in up to 96% of autistic individuals (Balasco et al., 2019; Baum et al., 2015; Billstedt et al., 2007; Crane et al., 2009; Marco et al., 2011). Moreover, the estimated prevalence of alexithymia, difficulties in recognizing, interpreting and distinguishing between one’s own emotions, is 50% in autistic individuals compared to 5% in non-autistic individuals (Kinnaird et al., 2019), further complicating effective stress regulation. The cumulative effect of these challenges faced by autistic individuals, together with a more stressful environment, can result in prolonged stress, posing significant health risks.

Evidently, there is a pressing need for therapeutic options that support autistic individuals in managing their daily stress levels (Bishop-Fitzpatrick et al., 2018). Previous efforts, such as the 8-week Mindfulness-Based Stress Reduction intervention, or the Mindfulness-Based Therapy–Autism Spectrum intervention, which target mind-body awareness and regulation of external stressors, have shown promising results for mental health (Beck et al., 2020; Braden et al., 2022; Sizoo & Kuiper, 2017; Spek et al., 2013). Yet, studies consistently show that autistic individuals often do not receive the help they need for their mental health problems (Cassidy et al., 2018; Jones et al., 2014; Mandy, 2022; Nicolaidis et al., 2013). One large survey study found the odds of having higher unmet mental healthcare needs were twice as high in autistic as compared with non-autistic adults (Nicolaidis et al., 2013). Several factors contribute to this gap, including significant barriers to accessing care (Camm-Crosbie et al., 2019; Mandy, 2022). A mixed methods study into the preferences of autistic individuals in mental health research and practice highlighted the need for accessible, evidence-based, self-management interventions that can be used independently, without relying on healthcare professionals (Benevides et al., 2020).

In this regard, mobile health (mHealth) applications may be particularly useful in managing daily stress for autistic individuals (Kim et al., 2018; Leung et al., 2021; Valencia et al., 2019; Zervogianni et al., 2020). mHealth can be employed in virtually any setting to provide real-time assistance (Kumm et al., 2022). It facilitates autonomy (Li et al., 2023), is predictive and structured in nature (Valencia et al., 2019), and can be implemented on a wide scale via smartphones. In the past decade, there has been a steep incline in the number of health apps available for the autistic population, with already more than 700 apps available in the Appstore as of 2017 (Kim et al., 2018). However, most of them lack empirical evidence (Kim et al., 2018). Moreover, mHealth intervention studies thus far mainly target children with autism, and focus on communication, social skills, or fine motor ability (Bharat et al., 2023; Leung et al., 2021; Moon et al., 2020; Valentine et al., 2020). To our knowledge, mHealth applications targeting perceived stress in adults with autism have hardly been part of rigorous scientific investigation. A recent telehealth-based mobile intervention aimed to support coping during stressful episodes using an “Emotional Support Plan” (Bal et al., 2024). However, this approach relies on stress recognition, while autistic individuals often fail to recognize these stress signals timely. Stress awareness is the first step toward effectively managing stress.

Stress Autism Mate (SAM), is an unguided, personalized mobile stress-monitoring application, developed in collaboration with autistic individuals, to support them in identifying and effectively managing their daily stress. One pre–post pilot study (*n* = 14) and a single-case design study (*n* = 34) showed positive effects on stress-related targets of the app, including perceived stress, coping self-efficacy, and quality of life (Hoeberichts et al., 2023, 2024). While these studies were important initial steps in evaluating the app, they were limited by small sample sizes, the absence of control groups, and a lack of randomization. Furthermore, potential bias may have arisen due to overlap in co-creation and study participation (Hoeberichts et al., 2023).

Building on these foundational findings, this randomized controlled trial (RCT) aimed to rigorously assess the effectiveness of the SAM application in achieving its intended goals: reducing perceived stress and improving mental well-being (primary outcomes), and coping self-efficacy (secondary outcome) of autistic adults. Also, we aimed to identify a potential dose-effect relationship between total days of application use and change scores for our primary outcome. Finally, we aimed to exploratively examine potential subgroups for whom the application was most effective. More specifically, we examined whether autism characteristics, sex, educational level, age, and co-occurring mental and/or somatic conditions influence the strength or direction of intervention effects. Based on the preliminary results by Hoeberichts et al. (2023, 2024), we hypothesized that 4 weeks of using the SAM application would yield a significant reduction in perceived stress, and a significant improvement of mental well-being and coping self-efficacy when compared to a waitlist control condition.

## Methods

### Study design

This is a two-arm, parallel RCT with a waitlist control condition. The study aimed to investigate whether 4 weeks use of the unguided SAM application yields a stronger reduction of perceived stress and improvement of mental well-being (primary outcomes), and coping self-efficacy (secondary outcome) than the waitlist control condition. Ethical approval was provided by the Scientific and Ethical Review Board of the Vrije Universiteit Amsterdam. This trial was pre-registered on 19 April 2023 via the ISRCTN Registry (trial registration number ISRCTN17394910).

### Procedures

#### Participants, recruitment, and eligibility criteria

Participants were recruited from the Netherlands Autism Register (NAR), a large cohort and database of individuals with autism in the Netherlands between 7 March and 12 April 2023. The NAR sends out yearly online questionnaires to collect data on the lives of autistic adults (through self-report or legal representatives) and autistic children up to age 16 (through parent report). For this study, a randomly drawn sample of 550 potential participants from the adult cohort (age > 16 years) was invited by email (Figure 2). The invitation contained detailed study- and app information and a link to an online informed consent form.

To be eligible for this study, participants ought to (a) be at least 16 years old; (b) have a formal diagnosis of autism spectrum disorder as verified when registering for the NAR; and (c) possess a smartphone or tablet with Internet connection. All participants had to provide digital informed consent upon registration for the study. Based on an alpha value of 0.05 and a power of 80%, 64 participants per arm were required (*n* = 128) to generate sufficient power to estimate a medium effect (*d* = 0.5) of SAM on perceived stress and mental well-being. Taking into account 20% dropout, the target number was 174 participants in total (87 per arm) (Karyotaki et al., 2021). This power calculation was based on the effect size of other mHealth interventions (Linardon et al., 2019; Moon et al., 2020; Versluis et al., 2016) and aligns with the effects of perceived stress and quality of life in pilot studies on SAM by Hoeberichts et al. (2023, 2024), who found small to medium effects on perceived stress and quality of life.

#### Randomization, blinding, and intervention allocation

After providing online informed consent, participants received an automated email with a link to the first assessment (T0). A built-in randomizer tool from Qualtrics (Qualtrics, Provo, UT) randomly assigned each participant filling out the baseline questionnaire to either the intervention condition or the waitlist control condition. Participants were informed on their allocated condition via email right after the baseline assessment. Due to the nature of the study, no blinding of the participants could be realized.

### Interventions

#### SAM

For 4 weeks, participants in the intervention group used the SAM mHealth application without guidance other than technical assistance by the research team. SAM is designed to reduce daily stress and improve mental well-being by aiding timely stress recognition in autistic individuals (Hoeberichts et al., 2023, 2024). In addition, the app aims to provide users with more control over their own stress levels through the use of coping tips.

SAM was co-created with 15 individuals with autism and their loved ones, and a project group of mental health researchers of the Netherlands Organization for applied scientific research (TNO), and clinicians of GGz Centraal, an organization for specialist mental health care in the center of the Netherlands, using a design thinking approach (Roberts et al., 2016). This is a user-centered approach to problem-solving that involves iterative stages of empathizing with users, defining problems, ideating, prototyping, and testing solutions to create innovative and effective designs.

Participants allocated to group 1 were instructed to use the SAM app daily for 4 weeks, until they received the follow-up questionnaire. They were provided with information on how the app works, how to install it, how to create an account, and how to adjust settings according to personal preferences (e.g. number of assessments per day, time slots for assessments, and selecting appropriate coping strategies for home versus away settings, as detailed in the next paragraph). In addition, they received a Frequently Asked Questions (FAQ) document and contact information for technical support.

**Figure 1.**
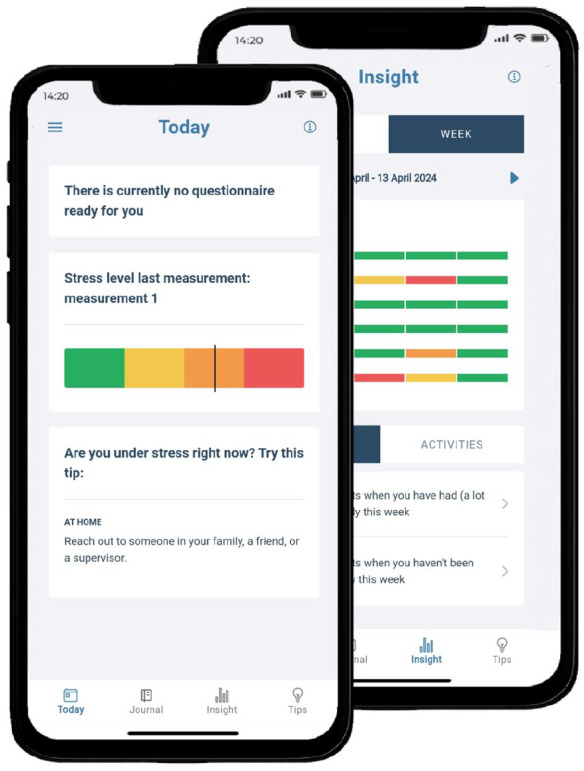
Screenshots of the SAM application overview pages. Two screenshots of the SAM application: The left screen shows the latest stress level, a button to the new assessment, and coping tips. The right screen displays a weekly overview of stress levels, color-coded from green to red. Users navigate pages using the bottom bar, with settings accessible via the top left icon.

Figure 1 provides screenshots of the application’s overview pages. To improve stress awareness, SAM prompts users to complete a three-minute questionnaire at 2, 3, or 4 fixed moments with a 4-h interval each day. During these assessments, users answer multiple choice questions about their recent activities and feelings. This is followed by stress-signaling questions, developed and tested through co-creation with autistic individuals, such as “Did you feel irritable?” and “Were you dreading activities on your schedule?.” Based on these questions, a validated algorithm calculates a sum score categorized as no stress, little stress, stress, or much stress, which is then summarized for the user. During the co-creation phase of SAM, this algorithm was tested with autistic individuals, who reported that 94.6% of the stress scores aligned with their self-assessed stress levels (Bonouvri et al., 2025). Users are then prompted to verify and reflect on how this summary matches their own perception. When stress is recorded, the app offers personalized coping tips depending on the user’s location (home or away), selected from a pre-identified list or self-invented by the user during registration. Example tips include “Go for a walk outside” or “Play a (video)game.” The app’s overview page displays stress levels throughout the day or week, with color-coded intensities (Figure 1). Discrepancies between app-assessed and self-perceived are also shown. As an additional feature, users can fill out diary entries. Layout preferences, number of assessments per day, and timing of the first assessment of the day can be altered anytime. Please see Hoeberichts et al. (2023) for more information on the application.

Around the time of the trial, the original SAM application was readily and freely accessible via app stores. However, we introduced a “research version” to distinguish study participants from regular users outside this trial. This version underwent minimal alterations to collect data on factors influencing stress in autistic adults. An overview of these modifications is provided in Table S1 of Supplementary Materials. Control participants were asked not to use the original SAM app. We checked (self-reported) use of the original SAM app during the T1 assessments.

#### Waitlist control

After the baseline assessment and randomization, participants in the waitlist control group were informed that they would gain access to the free SAM application in 4 weeks. In the meantime, participants in the control condition received no intervention and were asked not to use the regular SAM app. Care as usual was allowed in both study groups.

### Assessments

Demographic characteristics (e.g. age, sex assigned at birth, intellectual ability, educational attainment) were retrieved from the annual online assessment of the Netherlands Autism Register, which took place 1 month before the start of the current study. For this trial, all participants were assessed at two time points: at baseline, that is, just before the start of the intervention or waiting list (T0), and post-intervention, that is, 1 month after the start of the intervention (T1). Both assessments were completed online and included the same set of standardized questionnaires (described below). The second assessment was slightly more comprehensive, see below. Assessments took place between March 7 (first inclusion) and May 15 (latest follow-up assessment), 2023.

#### Primary outcomes

*Perceived Stress.* Perceived stress, our first primary outcome, was assessed using the 10-item version of the Perceived Stress Scale (PSS-10) (Cohen et al., 1983). The PSS-10 evaluates the degree to which an individual has perceived life as unpredictable, uncontrollable and overloading in the last month. Response options range from “Never” to “Very often,” coded from 0 to 4. Total PSS score ranges from 0 to 40, with a higher score indicating higher perceived stress. The PSS-10 is a widely used measure with good internal consistency in autistic samples (Cohen et al., 1983; Thoen et al., 2023). In this study, Cronbach’s alpha of baseline and post-intervention assessment was 0.868 and 0.899, respectively.

*Warwick-Edinburgh Mental Wellbeing Scale (WEMWBS)*. Mental well-being and its emotional, cognitive and psychological aspects were assessed using the Warwick-Edinburgh Mental Wellbeing Scale (Tennant et al., 2007). The scale comprises 14 positively phrased statements about feelings and thoughts in the last 2 weeks. Response options are scored on a 5-point Likert-type scale, ranging from 1 “None of the time” to 5 “all of the time.” Total WEMWBS score ranges from 14 to 70, with higher scores indicating a higher level of mental well-being. The scale has proven to be a largely unidimensional, reliable instrument with potential to predict health-related behaviors (Böhnke & Croudace, 2016). In this study, Cronbach’s alpha of baseline and post-intervention assessment was 0.900 and 0.914, respectively.

#### Secondary outcome

*Coping Self-Efficacy Scale (CSES).* Perceived coping efficacy, that is, the ability to perform coping behaviors, was assessed using the widely used 13-item version of the Coping Self-Efficacy Scale by Chesney et al. (2006). The CSES has three factors: use problem-focused coping, stop unpleasant emotions and thoughts, and get support from friends and family. Participants rate their confidence in performing adaptive coping behaviors on an 11-point scale (0 to 10, 0 = “I can’t at all” to 10 = “I can be sure”). Total CSES score ranges from 0 to 130, with higher scores indicating greater belief in one’s ability to perform coping behaviors. The CSES has good reliability and validity (Chesney et al., 2006). In this study, Cronbach’s alpha of baseline and post-intervention assessment was 0.917 and 0.928, respectively.

#### Application use, perceived effectiveness, and other stress-relieving interventions used

As a final part of the second assessment (T1), we included questions on SAM application use, perceived effectiveness, and other stress-relieving interventions used during the intervention period. The intervention group received the following questions: “Have you used the SAM app (almost) daily in the past 4 weeks?” (Yes/No), if no: “What is the reason that you have not used the SAM app daily in the past 4 weeks? Multiple answers can be selected.” (Technical problems with the app/Too little time for the app/Experiencing too little use of the app/The app actually increased my stress/Personal circumstances/Other.) The intervention group was also requested to rate the following statements on a 5-point Likert-type scale ranging from “Strongly agree” to “Strongly disagree,” and “cannot rate/not applicable”: “I gained more insight into my stress level,” “I gained more control over my stress level,” “I got stressed from using the app,” “I could relax better.” Finally, both the intervention and the control group were asked: “Have you ever used the original SAM app? (Yes/No),” if so, “Have you used this app in the past four weeks?” (Yes/No), “Have you followed a treatment or training in the past 4 weeks that made you experience less stress?” (Yes/No), if yes: “Can you indicate in the empty box below which treatment or training you followed?”

### Study—and intervention completers

Study completers were defined as participants who successfully completed both the baseline (T0) and post-intervention assessment (T1). To be categorized as an intervention completer, participants in the intervention group used the SAM app a minimum of 39 times. This threshold was established based on using the app twice daily for 4 weeks, at 70% adherence (28 days x 2 assessments/day x 0.7). We deemed this threshold of use feasible and necessary for achieving desired effects, based on previous studies into SAM (Hoeberichts et al., 2023, 2024). Control participants were considered “intervention completers” when they did not use the original SAM application while on the waitlist (self-reported).

### Statistical analyses

Data analysis was conducted using IBM SPSS Statistics (Version 27). Significance levels were set to *p* < 0.05. Selective attrition was examined by comparing baseline characteristics between study completers and study dropouts using t- and Chi-square tests. We also examined selective attrition by comparing those who adhered to the instruction of daily usage (at least 39 times usage of app) with those who did not. Baseline characteristics between study participants and those who declined to participate were compared using t- and Chi-square tests.

To estimate the effectiveness of the SAM app, we performed three separate hierarchical multiple regression analyses with post-intervention PSS, WEMWBS, and CSES scores as outcome variables and allocated condition (level 1) and baseline scores (level 2) as predictors. We adjusted for baseline scores to improve precision of intervention estimates and increase statistical power. Analyses were done on both intention-to-treat (ITT) principle and on Per-Protocol (PP) principle. For the ITT analyses, we exploited pre-scores and demographic characteristics (age, biological sex, ASQ scores, highest obtained education, employment status, relationship status) to impute missing data from study non-completers with 20 iterations, as we had 16% missing data (Graham et al., 2007). In the PP analysis, we included only those participants who used the app daily, as instructed (⩾39 times) (intervention group), and those who did not use the original SAM app during the 1-month intervention period (control group). Before conducting the PP analyses, we re-evaluated whether there were significant baseline differences between participants in the intervention and waitlist control that adhered to the protocol, because through exclusion of those who did not adhere, balance may have diminished.

(Pooled) Cohen’s d effect sizes were calculated by subtracting the intervention group’s mean scores from the mean score of the control group at post-intervention, and dividing this by the pooled standard deviations. After multiple imputation, Reliable Change Index (RCI) was calculated by dividing the difference between post-intervention and pre-intervention scores by the standard error of the difference between the two scores, with values greater than 1.96 indicating a statistically significant change between baseline and post-test. Chi-square tests were conducted to assess differences in the counts of reliably improved, reliably unchanged, and reliably deteriorated individuals between the intervention and control groups.

To investigate the dose effect relation between frequency of application use and changes in perceived stress (from T0—T1), we performed Pearson’s bivariate correlations. Explorative post hoc subgroup analyses were conducted to find moderators of intervention effects by adding interaction terms between intervention group and predictors baseline scores (standardized), age (standardized), biological sex (categorical), educational level (categorical), Autism Quotient Score (standardized), and co-occurring mental and/or somatic diagnoses (categorical) to the regression models. As running multiple tests can increase the risk of detecting spurious association, and these subgroup analyses were exploratory, the significance level was set to a more conservative *p* < 0.01 for these analyses, decreasing the risk of Type I errors (Feise, 2002; Perneger, 1998).

### Community involvement statement

The SAM application was developed in co-creation with autistic clients of GGz Centraal, see Hoeberichts et al. (2023). Autistic individuals were involved in testing the flow, duration, and clarity of the online measurements of this trial. In addition, insights from conversations with SAM users and autistic clients of GGz Centraal were referenced during the interpretation of the findings, ensuring that the perspectives of autistic individuals informed the study outcomes. Formal involvement of autistic advisors in the study design or structured result interpretation sessions was not undertaken.

## Results

### Participants and study flow

**Figure 2.**
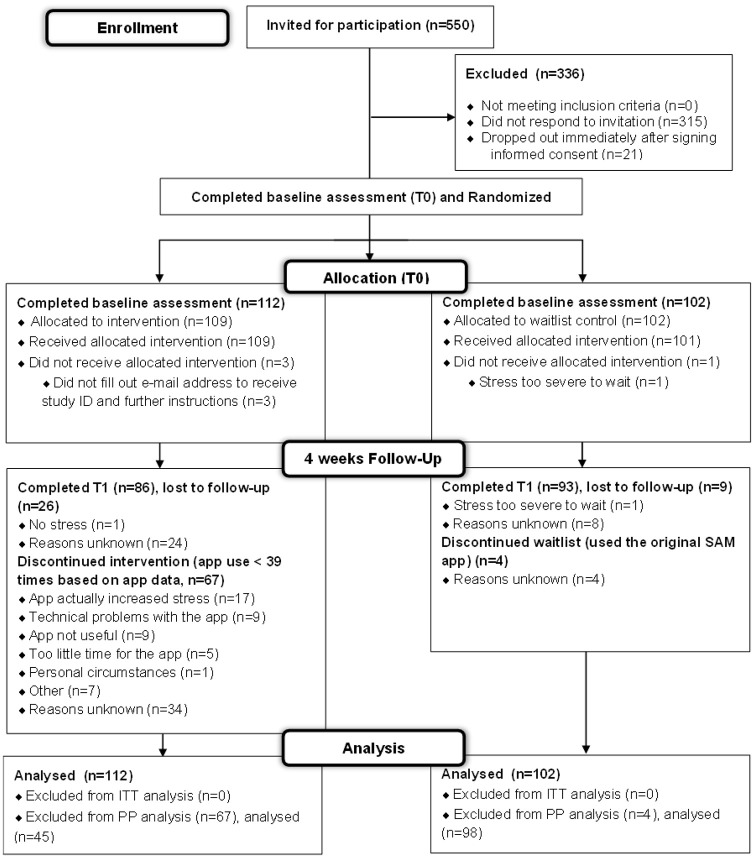
CONSORT flow diagram. Intent-to-treat (ITT), Per-Protocol (PP). In this flowchart, intervention dropout was based solely on our pre-defined cut off for frequency of use. An overview of data on self-reported app use and app use as collected through application data can be found in Supplementary Materials (Table S4).

See Table 1 for participants characteristics of both groups as originally randomized, and Figure 2 for study flow.

**Table 1.** Demographic characteristics of the two study arms as randomized.

Variables	Intervention *n*(%) or *M*(*SD*) (*n* = 112)	Waitlist control *n*(%) or *M*(*SD*) (*n* = 102)
Age	49.6 (12.0)	48.6 (13.9)
Sex assigned at birth, Female	73 (65.2)	69 (67.6)
Autism Quotient – Short (AQ-Short) by Hoekstra et al. (2011)	84.7 (10.2)	83.8 (10.7)
Intellectual ability^a^		
IQ <86	8 (7.1)	13 (12.7)
IQ 86–115	23 (20.5)	22 (21.6)
IQ >116	79 (70.5)	61 (59.8)
Educational attainment		
Low	11 (9.8)	10 (9.8)
Middle	41 (36.6)	33 (32.4)
High	58 (51.8)	58 (56.9)
Dutch ethnicity	107 (95.5)	92 (90.2)
Age of diagnosis	39.1 (15.0)	39.0 (13.9)
Co-occurring mental health condition	52 (46.4)	50 (49.0)
Romantic relationship		
Yes	67 (59.8)	59 (57.8)
No	44 (39.3)	41 (40.2)
Don’t know/uncertain	1 (0.9)	2 (2.0)
Paid job	54 (48.2)	50 (49.0)
Living situation		
Living with partner and/or children	60 (53.6)	46 (45.1)
Independently alone	34 (30.4)	35 (34.3)
Independently alone (some housing assistance)	11 (9.8)	9 (8.8)
Living with partner and/or children (housing assistance)	3 (2.7)	3 (2.9)
Living in form of housing with supervision and/or care	2 (1.8)	1 (1.0)
Living with parents/caretakers	0 (0.0)	6 (5.9)
Healthcare facility	1 (0.9)	1 (1.0)
Other	1 (0.9)	1 (1.0)
Outcomes^b^		
Perceived Stress (PSS-10)	20.8 (6.2)	20.7 (6.4)
Mental well-being (WEMWBS)	43.1 (7.8)	41.5 (8.9)
Coping Self-Efficacy (CSES)	53.0 (22.6)	54.8 (23.6)

a Intellectual ability was self-reported: 49.1% of the intervention group and 47.6% of the control group based their IQ on a test score, compared to 42.0% and 39.8% who estimated their IQ themselves.

b Perceived Stress Scale; Warwick-Edinburgh Mental Wellbeing Scale; Coping Self-Efficacy Scale.

As group disbalances may arise in PP groups, we examined whether significant differences existed between these groups. No significant group differences were observed in any of the characteristics, except for self-reported IQ (χ^2^ = 7.58; *p* = 0.023), with relatively higher IQ scores in the intervention group.

When comparing baseline characteristics between study participants and the group that declined to participate (*n* = 315), we found the average AQ score of study participants to be higher (*M* study participants = 84.3, *M* difference = 2.6, *t*(542) = 2.81, *p* = 0.005).

Our analysis of selective attrition showed no differences between study dropouts and completers on most variables (age, sex, education, employment, co-occurring diagnoses, baseline scores), except for age of autism diagnosis: dropouts were diagnosed at a younger age than completers (*M* diff = 7.8, *SE* diff = 3.3, *t*(106) = 2.32, *p* = 0.022). When comparing those who used the app at least 39 times versus those who did, we found that the latter had higher CSES scores at baseline (*M* diff = 11.3, *SE* diff = 4.9, *t*(84) = 2.27, *p* = 0.026). Study—and intervention completion data and data on other stress-related interventions used during the study can be found in Supplementary Materials (Table S2 and S3, respectively).

### Main analyses

#### Primary outcomes

See Table 2 for the results of the main hierarchical regression analyses, investigating intervention effects. After controlling for baseline scores, ITT analyses showed significant intervention effects for both perceived stress and mental well-being. Post-intervention, the intervention group reported significantly lower levels of perceived stress (*d* = 0.27, *p* = 0.003) and significantly higher mental well-being (*d* = 0.35, *p* = 0.013) compared to the waitlist control group. When only including those who adhered to their assigned condition (PP analyses), the intervention effects were slightly larger for both perceived stress (*d* = 0.35, *p* < 0.001) and mental well-being (*d* = 0.39, *p* = 0.005). Reliable Change Indexes for all outcomes are displayed in Table 3.

#### Secondary outcome

ITT analyses showed a significant intervention effect for coping self-efficacy (*d* = 0.16, *p* = 0.007) (Table 2). Post-intervention, the intervention group reported significantly better coping self-efficacy than the waitlist control group. When only including those who adhered to their assigned condition (PP analyses), the effect decreased (*d* = 0.11, *p* = 0.020).

**Table 2.** Intervention effect estimates for the intervention group compared to the waitlist control group.

Intent-to-treat analysis (*n* = 214)					
Outcome	*B* (*SE*)		*t*	*p*	Cohen’s *d*
Primary					
Perceived stress	–1.92 (0.64)		–2.99	**.003**	0.27
Mental well-being	1.92 (0.77)		2.48	**.013**	0.35
Secondary					
Coping Self-Efficacy	5.28 (1.97)		13.29	**.007**	0.16
Per-Protocol analysis (*n* = 143)					
Outcome	*B (SE)*	Beta	*t*	*p*	Cohen’s *d*
Primary					
Perceived stress	–3.31 (0.81)	0.23	–4.07	**<0.001**	0.35
Mental well-being	2.81 (0.99)	0.15	2.84	**0.005**	0.39
Secondary					
Coping Self-Efficacy	8.03 (2.57)	0.13	2.35	**0.020**	0.11

Intent-to-treat analyses include all participants as originally randomized. Per-Protocol analyses include only those participants who used the app daily, as instructed (⩾ 39 times) (intervention group), and those who did not use the original SAM app during the 1-month intervention period (control group). For improved precision of estimates, we controlled for baseline scores.

Significant findings (*p* < 0.05) are presented in bold.

**Table 3.** Reliable Change Indexes of the outcomes at T1 (post-intervention).

	Intervention *n*(%)	Control *n*(%)	Test statistic χ^2^	*df*	*p*-value
PSS—Reliable Change			6.34	2	**.042**
Reliably improved	16 (14.3)	7 (6.9)			
Reliably unchanged	93 (83.0)	86 (84.3)			
Reliably deteriorated	3 (2.7)	9 (8.8)			
WEMWBS—Reliable Change			4.00	2	.135
Reliably improved	7 (6.3)	7 (6.9)			
Reliably unchanged	100 (89.3)	83 (81.4)			
Reliably deteriorated	5 (4.5)	12 (11.8)			
CSES—Reliable Change			6.88	2	**.032**
Reliably improved	10 (8.9)	7 (6.9)			
Reliably unchanged	99 (88.4)	83 (81.4)			
Reliably deteriorated	3 (2.7)	12 (11.8)			

PSS: Perceived Stress Scale; WEMWBS: Warwick-Edinburgh Mental Wellbeing Scale; CSES: Coping Self-Efficacy Scale. Pooled counts after MI are displayed. Significant findings (*p* < 0.05) are presented in bold.

#### Subgroup analyses—moderators of effect

The main intervention effect of condition on post-intervention scores of PSS, WEMWBS and CSES was not significantly moderated by any of the preselected variables (Table S5, Supplementary Materials). No subgroups could be identified. However, the sample sizes within subgroups may have been too small to detect significant differences.

#### Dose-response analysis

Linearity checks revealed a linear relationship between app use frequency and change scores of our primary outcomes. App use frequency was significantly negatively correlated with change scores for perceived stress (*r* = –0.293, *p* = 0.013), indicating that more frequent app usage was associated with a stronger decrease in perceived stress. App use frequency was positively correlated with change scores for mental well-being (*r* = 0.380, *p* = 0.001), and not significantly correlated with change scores for coping self-efficacy (*r* = 0.058, *p* = 0.633). Data on frequency of app use by the intervention group can be found in Table S6 of Supplementary Materials.

### Perceived effectiveness

See Table 4 below for the perceived effects of the intervention, as reported by the intervention group. Participants who reported experiencing increased stress from using the app (*n* = 48) used the app significantly less frequently (*M* = 42.6, *SD* = 33.3) than those who did not report feeling stressed (*n* = 38, *M* = 78.1, *SD* = 45.5). The mean difference in usage was 35.6 times (*SE* = 10.1), with a statistically significant difference between the groups, *t*(68) = 3.54, *p* < 0.01. No sampling differences between these two groups were found for aforementioned characteristics, baseline scores, or change scores.

**Table 4.** Statements on self-perceived effects of the intervention.

Statement	(Strongly) Disagree *n*(%)	Neither agree or disagree *n*(%)	(Strongly) agree *n*(%)	Cannot judge/NA *n*(%)	Mean (*SD*)
I gained more insight into my stress level	22 (19.6)	7 (6.3)	45 (40.1)	12 (10.7)	3.45 (1.45)
I gained more control over my stress level	37 (33.0)	18 (16.1)	17 (15.2)	14 (12.5)	2.46 (1.30)
I got stressed from using the app	22 (19.6)	9 (8.0)	48 (42.9)	7 (6.3)	2.9 (1.51)
I could relax better	41 (36.6)	18 (16.1)	14 (12.5)	13 (11.6)	4.0 (1.26)

Responses range from 1 (*Strongly disagree*) to 5 (*Strongly agree*) are coded from 1 to 5, with higher scores indicating stronger agreement. This table is based on data from the entire intervention group, including those who quit the intervention. Valid percentages are displayed.

## Discussion

In this RCT, we aimed to assess the effectiveness of SAM, a mobile health application designed in co-creation with autistic individuals, to aid individuals with autism in managing daily stress. Intent-to-treat analyses revealed that participants in the intervention group experienced a greater reduction in perceived stress, improvement in mental well-being and an increase in coping self-efficacy compared to the waitlist control group. Adherence to daily use of the app was 40.2%. PP analyses showed slightly larger effects for perceived stress and mental well-being, and a decrease in the intervention effect for coping self-efficacy. The Reliable Change Index (RCI) showed that significantly more intervention participants reliably improved in perceived stress scores, and more control participants reliably deteriorated on all three outcomes. Notably, 40.1% indicated to have gained more insight in their stress levels, although 43% of the intervention group indicated increased stress due to the app. More frequent app use was associated with greater reduction in perceived stress and greater improvement of mental well-being. No subgroups for whom the intervention was either more or less effective could be identified.

### Potential mechanisms of effect

The results of this RCT support the preliminary results from studies by Hoeberichts et al. (2023, 2024), who found that SAM positively affected perceived stress, coping self-efficacy, psychological well-being, and self-rated quality of life of autistic adults. Here, we propose several potential mechanisms for the observed reduced perceived stress and improved well-being in this study. First, the application promotes self-monitoring, a popular strategy in stress-management apps (Alhasani et al., 2022). Self-monitoring has long been recognized for its ability to enhance self-awareness and facilitate positive behavioral development (Alhasani et al., 2022; Korotitsch & Nelson-Gray, 1999). The underlying theory suggests that when individuals are prompted to reflect on their momentary states, experiences, behaviors, and thoughts in close proximity to their occurrence, it can promote mindfulness and create opportunities for personal transformation, such as altering the environment to better fit the needs of an individual (Chen et al., 2017). In line with this, amelioration of alexithymia or improvement of interoception could be a plausible mechanism that may have contributed to the observed effects on our primary outcomes (Cameron et al., 2014). Indeed, many study participants (40.1%) indicated that their stress awareness increased using the app.

Another potential mechanism of effect could have arisen from the app’s personalized coping tips following elevated stress levels (e.g. “talk with a loved one” or “go for a walk”). These coping tips align with various coping strategies, including seeking social support, leisure, and exercise, all of which are shown to positively impact well-being in autistic adults (Charlton et al., 2023; García-Villamisar & Dattilo, 2010; Hallett, 2019). Coping is an essential element of the app, as it is applied when the app detects elevated stress levels, with the aim to help reduce this stress. However, similar to Hoeberichts et al. (2024), we found the smallest effect for coping self-efficacy with the CSES. In addition, only 15.2% of participants indicated they gained more control over their stress. This suggests that the SAM app falls short in achieving one of its intended goals, leaving space for further enhancements. Interestingly, participants who did not adhere to the intervention had significantly higher baseline CSES scores, indicating greater coping self-efficacy. This may suggest that individuals who perceive themselves as more capable of managing stress may be less motivated to engage with external stress-management tools, such as the SAM app.

When asked to retrospectively reflect on their experience with the app, 42.9% of participants indicated that they got stressed from the app, contrary to its primary goal to reduce stress. While this finding comes from a single, non-standardized question and should be interpreted with caution, two explanations are discussed here. The stress reaction toward the app could be an undesirable side effect of the intervention, such as technostress caused by sensory stimulation from push notifications or added demands to one’s already stressful daily obligations (Cao et al., 2020; Schroeder et al., 2021). It may also be a necessary element for SAM to be effective (accompanying the increased stress awareness). According to the Social Cognitive Theory of Self-Regulation by Bandura (1991), becoming aware of one’s current (distressed) state and detecting a discrepancy between this state and the desired state (not stressed) can elicit negative emotions like distress, anxiety or frustration. Whether this negative discrepancy between the desired and current state is motivating or discouraging for altering one’s behavior is partly determined by perceived coping self-efficacy (Bandura, 1991), once again underscoring the importance of enhancing this aspect through the app.

As anticipated, the PP analyses, which included only those individuals who strictly adhered to the study protocol, revealed a moderately stronger effect on the primary outcomes compared to ITT analyses. This finding aligns with expectations, as participants who made little or no use of the app would naturally derive fewer effects, and is consistent with our finding that more frequent app usage yields stronger effects. The protocol threshold of 39 times use may have been overly strict though, as even lower levels of usage appear to be beneficial.

Across all outcomes, we observed a pattern of significantly more participants in the waitlist control group experiencing deterioration compared to the intervention group. This might suggest a potential effectiveness of the SAM intervention in preventing or mitigating declines in mental well-being. The findings might also emphasize the challenges faced by individuals on a waitlist (Patterson et al., 2016), highlighting the importance of timely mental health interventions.

### Clinical implications

According to the conventional Cohen’s *d* benchmarks (Cohen, 1988), the intervention effects on perceived stress, mental well-being, and coping self-efficacy were small. Yet, interpreting standardized mean effect sizes is a challenging endeavor that is highly dependent on the specific field of study and the nature of the conducted trial (Schuetze & Yan, 2023). In this study, intervention effects were present even though participants using SAM did not receive any professional support from researchers or clinicians other than technical support. This seems to indicate that SAM may have small positive effects in real-world conditions outside clinical practice, making it a potentially useful and accessible tool for autistic adults to monitor and alleviate their stress in everyday life.

The effects of SAM are difficult to compare with effects observed in other studies, because mHealth literature in the autistic population is generally very scarce (Kim et al., 2018; Lecomte et al., 2020; Leung et al., 2021; Moon et al., 2020). When compared to meta-analyses about mHealth effectiveness in other (clinical) populations, the SAM app seems somewhat less effective in reducing perceived stress and improving mental well-being: Versluis et al. (2016) found a small effect size (g = 0.40) for the effect of Ecological Momentary Assessment (EMA) interventions on perceived stress (PSS) in both clinical (e.g. with anxiety—and depressive disorders) and community samples (199 participants across five studies). Yet, two of the included studies were pre–post studies, potentially inflating the ES. Linardon et al. (2019) reported a Hedges g of 0.47 for mental health smartphone apps on stress compared to waitlist control, based on 20 RCTs involving both symptomatic/at-risk samples and non-symptomatic samples.

All in all, the observed effects have some important clinical implications. Since stress is a transdiagnostic factor, an mHealth app targeting stress may further contribute to the prevention of psychopathology and physical illness in autistic individuals. SAM might serve as a valuable add-on in psychotherapy when clinicians identify difficulties with stress regulation, or may be used as a stand-alone intervention. Given SAM’s availability in 33 countries across Europe, Canada, New Zealand, and Australia, in seven languages, with free access and an autism-friendly helpdesk, it is widely accessible for users.

### Strengths and limitations

This study demonstrated notable strengths, including a large sample size of 214 autistic adults, which ensured sufficient statistical power for detecting subtle intervention effects. SAM was co-created with autistic individuals, which is important as it enhances the app’s relevance and usability by addressing the specific needs and preferences of the target population. In addition, the study exhibited a relatively low study dropout rate, with only 16% of participants discontinuing the study after their condition allocation. Although literature on (dropout in) mHealth interventions for autistic individuals is still scarce, a 16% study dropout sharply contrasts with the high attrition rates commonly observed in studies evaluating mobile health interventions in other clinical samples (Linardon et al., 2024; Torous et al., 2020).

Despite these strengths, the study also encountered limitations. First, 59.8% of participants discontinued the intervention based on our pre-defined cut-off. However, this intervention completer cut-off may have been too stringent, as 50.9% of participants reported to have used the app almost daily (Table S4). A large meta-analysis with 89 RCTs studying attrition in meditation apps, found the number of times the app was used to be between 3.4 and 51 (*SD*s between 2 and 44), whereas the average was 48.2 times in our study, suggesting relatively high engagement. Moreover, it must be noted that high dropout rates are common in mHealth studies, where participants are generally more likely to discontinue than to complete the intervention (Amagai et al., 2022; Ross et al., 2016). Other mHealth studies have found similar but slightly lower attrition rates, with Bremer and Sarker (2023) reporting an average rate of 39.56% across 21 studies with various clinical samples, and Linardon (2023) reporting 38.7% attrition across 6123 participants participating in larger trials (*n* > 100 per group) like our own RCT. In real-world usage of mHealth, retention rates are only 4.1% and 6.3% for mindfulness/meditation and tracker apps, respectively (Baumel et al., 2019). In-app support systems such as peer or coach support, or gamification elements might have improved retention (Amagai et al., 2022; Bremer and Sarker, 2023; Jakob et al., 2022), but could increase the costs and accessibility of SAM.

Second, the relatively short follow-up period of 4 weeks may not have been sufficient for the full effects of SAM to be manifested or captured. The PSS at T1 assessed stress levels over the same 1-month period during which the app was used, limiting our ability to capture changes that may have manifested beyond this timeframe. The median time to form new health-related habits in the general population is estimated to be approximately 2 months (Singh et al., 2024). It is possible that participants required more time to internalize the process of monitoring their mental and physical states, detecting stress, and employing effective coping techniques.

Finally, it should be acknowledged that Dutch, female, intellectually able adults with autism who were diagnosed in adulthood were overrepresented in our study, potentially limiting the generalizability of our findings.

### Suggestions for future research

We suggest several areas for future research on SAM. First, exploring ways to enhance the app’s coping support could improve its effectiveness. Recent work by Bal et al. (2024), who developed an “Emotional Support Plan” that autistic adults can personalize for use during moments of distress, could serve as an example. In addition, the high percentage of increased stress due to app use needs further investigation. Qualitative, participatory research such as in-depth interviews or focus groups could be employed to address these concerns and capture valuable insights into individual experiences helping to guide further improvements. Investigating the app’s potential as a complementary, guided tool for psychotherapy instead of a stand-alone intervention also deserves further investigation. Although no moderators of effect/subgroups could be identified in this study—likely due to small sample sizes within subgroups—the app appears to be more suited for some individuals than for others. Identifying these individuals can inform health care professionals on which clients could benefit from SAM. Finally, a longer follow-up period in future studies (e.g. 2 and 6 months) would provide valuable insights into the sustainability and long-term impact of SAM. By addressing these research gaps, future studies can ensure a more effective approach to stress reduction for individuals with autism.

## Conclusion

Our results suggest that SAM can potentially serve as a scalable tool for reducing perceived stress and improving mental well-being in some autistic adults. However, several of our findings warrant further investigation: 85% of participants did not show a reliable change in their outcomes and 42.9% indicated to get stressed from the app, contrary to its primary goal. SAM may prove particularly effective for a yet-to-be identified specific subgroup of individuals. Although we were unable to pinpoint this subgroup, the observed dose-response relationship suggests that those who found the app helpful and engaged with it more frequently, experienced greater stress reduction and improved mental well-being. Moving forward, there is still room for improvements of the application, particularly for coping self-efficacy.

## Supplemental Material

sj-docx-1-aut-10.1177_13623613251346885 – Supplemental material for A randomized controlled trial into the effectiveness of a mobile health application (SAM) to reduce stress and improve well-being in autistic adults Supplemental material, sj-docx-1-aut-10.1177_13623613251346885 for A randomized controlled trial into the effectiveness of a mobile health application (SAM) to reduce stress and improve well-being in autistic adults by Kirsten L Spaargaren, Yvette Roke, Sander M Begeer, Annemieke van Straten, Heleen Riper, Kirstin Greaves-Lord and Anke M Scheeren in Autism

## Notes

1. Person-first and identity-first language are used interchangeably throughout this paper, given the variability in preferred terminology among individuals with autism (Buijsman et al., 2022; Kenny et al., 2015).
