# Supplementary material for: A randomized controlled trial into the effectiveness of a mobile health application (SAM) to reduce stress and improve well-being in autistic adults
Source: Autism. 2025 Jun 26;29(10):2588–603. doi: 10.1177/13623613251346885 (PMC12417602; doi:10.1177/13623613251346885)
Supplement: sj-docx-1-aut-10.1177_13623613251346885 – Supplemental material for A randomized controlled trial into the effectiveness of a mobile health application (SAM) to reduce stress and improve well-being in autistic adults [file sj-docx-1-aut-10.1177_13623613251346885.docx]

**Supplementary materials**

**Table S1.**
 *Additional questions posed in the research version of SAM*

|  | Question | Response options |
| --- | --- | --- |
|  | In the past four hours, I was mainly | Alone  With partner  With family or friends  With other people |
|  | In the past four hours, I was mainly with | People with autism  People without autism  A mixture of people with and without autism  Not applicable, because I was alone |
|  | In the past four hours, I was mainly | At home  Not at home |
|  | In the past four hours, I was mainly | Inside  Outside |
|  | In the past four hours,  I was physically active | Yes, strongly  Yes, a little  No |
|  | In the past four hours,  I was engaged in my hobbies or interests | Yes, strongly  Yes, a little  No |
|  | In the past four hours, I could completely be myself | Yes, strongly  Yes, a little  No |
|  | In the past four hours, I was bored | Yes, strongly  Yes, a little  No |

*Note.* In the research version of the SAM application, participants were asked eight additional questions during each assessment. While this data is intended for a separate research project, it is included here because it was part of the version used by our study participants.

**Table S2.**
*An overview of study – and intervention completers*

|  |  | Intervention completer | | |
| --- | --- | --- | --- | --- |
|  |  | Yes n(%) | No n(%) | Total n(%) |
| Study completer | Yes n(%) *Intervention Control* | 133 (62.1) 44 89 | 46 (21.5) 42 4 | 179 (83.6) 86 93 |
|  | No n(%) *Intervention Control* | 10 (4.7) 1 9 | 25 (11.7) 25 0 | 35 (16.4) 26 9 |
|  | Total n(%) *Intervention Control* | 143 (66.8) 45 98 | 71 (33.2) 67 4 | 214 (100.0) |

*Note.* Study completers are those participants who completed both the baseline and post-intervention assessment. Intervention completers are those participants in the intervention group who used SAM at least 39 times (based on app data), and those in the control group who did not use the original SAM app during the intervention period (based on self-report, since app data from the original app was not available).

**Table S3.**

*Other stress-related interventions used during 1-month intervention period*

| Type of stress-reducing intervention | Intervention | Waitlist control | Total |
| --- | --- | --- | --- |
| Psychological support | 10 | 13 | 23 |
| Psychomotor & Psychosomatic therapy & Physical Exercise | 4 | 1 | 5 |
| Meditation-related approaches | 2 | 1 | 3 |
| Total | 16 | 15 | 31 |

*Note.* Total number of interventions exceeds 25, since some participants received multiple interventions during the intervention period.

**Table S4.**

*Self-reported app use and app use as collected through application data, congruences and discrepancies*

| (Likely) daily use of SAM | | |
| --- | --- | --- |
|  | n | % |
| Self-reported daily use and app data suggests at least 39 times usage (congruence) | 41 | 36.6 |
| Self-reported daily use but app data suggests less than 39 times usage (discrepancy) | 9 | 8.0 |
| No self-reported daily usage but app data suggests at least 39 times usage (discrepancy) | 3 | 2.7 |
| Likely daily use, but 1 missing variable to verify | 4 | 3.6 |
| Total (likely) daily use of SAM | 57 | 50.9 |
| No (likely) daily use of SAM | | |
| No self-reported daily use and app data suggests less than 39 times usage (congruence) | 17 | 15.2 |
| Likely no daily use, but missing data for one or both variables | 38 | 33.9 |
| Total (likely) no daily use of SAM | 55 | 49.1 |

*Note*. Discrepancy and congruence indicate a discrepancy or congruence between self-reported daily use and daily use according to our pre-defined cut-off. PP analyses were strictly based on app data.

**Table S5.***Moderation analyses of main effect*

| Model | Variables | B | SE | t | p |
| --- | --- | --- | --- | --- | --- |
|  | OUTCOME 1: PERCEIVED STRESS | | | |  |
| Model 1 | CONDITION BASELINE SCORE CONDITION x BASELINE SCORE | -0.284 -0.785 0.026 | 0.085 0.154 0.98 | -2.996 -5.105 0.261 | **.003* <.001**** .794 |
| Model 2^a^ | CONDITION  ASQ SCORES CONDITION x ASQ SCORE | -0.283  0.065  -0.038 | -0.095  0.159  0.098 | -2.987  0.411  -0.385 | **.003***  .658  .700 |
| Model 3^a^ | CONDITION  BIOLOGICAL SEX CONDITION x BIOLOGICAL SEX | -0.587  -0.370  0.184 | 0.340  0.316  0.197 | -1.726  -1.174  0.930 | .085  .241  .352 |
| Model 4^a^ | CONDITION  EDUCATIONAL LEVEL CONDITION x EDUCATIONAL LEVEL | -0.285  0.101  -0.059 | 0.095  0.157  0.098 | -3.000  0.641  -0.606 | **.003***  .522  .545 |
| Model 5^a^ | CONDITION  AGE CONDITION x AGE | -0.287  -0.087  0.099 | 0.095  0.098  0.159 | -3.021  -0.886  0.624 | **.003***  .376  .533 |
| Model 6^a^ | CONDITION  CO-OCCURING DIAGNOSES CONDITION x CO-OCCURRING DIAGNOSES | -0.072  0.242  -0.117 | 0.317  0.320  0.200 | -0.322  0.758  -0.586 | .748  .449  .558 |
| Model | Variables | B | SE | t | p |
|  | OUTCOME 2: MENTAL WELLBEING | | | |  |
| Model 1 | CONDITION  BASELINE SCORE CONDITION x BASELINE SCORE | 0.218 -0.997 0.138 | 0.088  0.140  0.088 | 2.485  -7.123  1.574 | .013  **<0.001**** .116 |
| Model 2^a^ | CONDITION  ASQ SCORE CONDITION x ASQ SCORE | 0.217  0.095  -0.068 | 0.088  0.154  0.093 | 2.465  0.621  0.734 | .014  .535  .463 |
| Model 3^a^ | CONDITION  BIOLOGICAL SEX CONDITION x BIOLOGICAL SEX | 0.159  -0.024  0.035 | 0.321  0.299  0.188 | 0.494  -0.082  0.188 | .621  .935  .851 |
| Model 4^a^ | CONDITION  EDUCATIONAL LEVEL CONDITION x EDUCATIONAL LEVEL | 0.221  0.035  -0.060 | 0.088  0.149  0.091 | 2.512  0.237  -0.656 | .012  .813  .512 |
| Model 5^a^ | CONDITION  AGE CONDITION x AGE | 0.218  -0.788  0.059 | 0.088  0.087  0.144 | 2.464  -0.456  0.413 | .014  .648  .680 |
| Model 6^a^ | CONDITION  CO-OCCURRING DIAGNOSES CONDITION x CO-OCCURRING DIAGNOSES | 0.374  0.064  -0.099 | 0.293  0.300  0.183 | 1.274  0.212  -0.541 | .203  .833  .589 |

*Note.* **significant at the <.001 level; *significant at the <.01 level; ^a^model adjusted for baseline levels of outcome

**Table S6.**

*Frequency of application use by intervention group*

| Category | Frequency (percentage) |
| --- | --- |
| 0 – 25 times | 35 (31.3) |
| 26 – 50 times | 20 (17.9) |
| 51 – 75 times | 9 (8.0) |
| 76 – 100 times | 9 (8.0) |
| 101 – 125 times | 7 (6.3) |
| 126 – 150 times | 2 (1.8) |
| 151 – 175 times | 2 (1.8) |
| 176 – 200 times | 2 (1.8) |
| Adherence threshold: 0 – 38 times | 41 (36.6) |
| Adherence threshold: ≥39 times | 45 (40.2) |
| Total | 86 (76.8) |

*Note.* The number of times participants in the intervention group used the application during the four weeks intervention period. A summary is provided for usage below and above the threshold of ≥39 times for adherence. Percentages don’t add up to 100 due to missing data.
